# Mustard Gas Exposure Actuates SMAD2/3 Signaling to Promote Myofibroblast Generation in the Cornea

**DOI:** 10.3390/cells12111533

**Published:** 2023-06-02

**Authors:** Nishant R. Sinha, Ratnakar Tripathi, Praveen K. Balne, Laila Suleiman, Katherine Simkins, Shyam S. Chaurasia, Rajiv R. Mohan

**Affiliations:** 1 Departments of Veterinary Medicine & Surgery and Biomedical Sciences, University of Missouri, Columbia, MO 65211, USA; 2 Harry S. Truman Memorial Veterans’ Hospital, Columbia, MO 65201, USA; 3 Ocular Immunology and Angiogenesis Lab, Department of Ophthalmology & Visual Sciences, Froedtert & Medical College of Wisconsin Eye Institute, Milwaukee, WI 53226, USA; 4 Department of Cell Biology, Neurobiology and Anatomy, Medical College of Wisconsin, Milwaukee, WI 53226, USA; 5 Mason Eye Institute, School of Medicine, University of Missouri, Columbia, MO 65212, USA

**Keywords:** sulfur mustard, mustard gas keratopathy, cornea, myofibroblasts, SMADs, haze

## Abstract

Sulfur mustard gas (SM) is a vesicating and alkylating agent used as a chemical weapon in many mass-casualty incidents since World War I. Ocular injuries were reported in >90% of exposed victims. The mechanisms underlying SM-induced blindness remain elusive. This study tested the hypothesis that SM-induced corneal fibrosis occurs due to the generation of myofibroblasts from resident fibroblasts via the SMAD2/3 signaling pathway in rabbit eyes in vivo and primary human corneal fibroblasts (hCSFs) isolated from donor corneas in vitro. Fifty-four New Zealand White Rabbits were divided into three groups (Naïve, Vehicle, SM-Vapor treated). The SM-Vapor group was exposed to SM at 200 mg-min/m3 for 8 min at the MRI Global facility. Rabbit corneas were collected on day 3, day 7, and day 14 for immunohistochemistry, RNA, and protein lysates. SM caused a significant increase in SMAD2/3, pSMAD, and ɑSMA expression on day 3, day 7, and day 14 in rabbit corneas. For mechanistic studies, hCSFs were treated with nitrogen mustard (NM) or NM + SIS3 (SMAD3-specific inhibitor) and collected at 30 m, 8 h, 24 h, 48 h, and 72 h. NM significantly increased TGFβ, pSMAD3, and SMAD2/3 levels. On the contrary, inhibition of SMAD2/3 signaling by SIS3 treatment significantly reduced SMAD2/3, pSMAD3, and ɑSMA expression in hCSFs. We conclude that SMAD2/3 signaling appears to play a vital role in myofibroblast formation in the cornea following mustard gas exposure.

## 1. Introduction

Sulfur mustard gas (SM) is a chemical warfare agent used in many wars and conflicts since its first use in World War I [1,2,3,4,5,6]. Sulfur mustard is the most abundantly stockpiled chemical warfare agent worldwide due to its easy production, easy deployment, and strong vesicant properties. The recent employment of SM in terrorist activities and the Syrian civil war is a significant concern. SM is known to cause severe injuries to the skin, eyes, and lungs, with long-term complications. For instance, about 50% of the 100,000 Iranians exposed to SM during the Iraq–Iran war from 1980–1988 suffered chronic respiratory, skin, and eye complications [1,2,3,4,5,6]. Unfortunately, 90% of SM gas-exposed victims experience acute and chronic ocular symptoms 30+ years after exposure [4,7,8].

SM exposure to the eyes causes severe corneal injury, ocular pain, irritation, and significant vision impairment [9]. In some cases, ocular complications persisted several months to years after the initial exposure [4,7,10,11]. The ocular pathology resulting from SM exposure is often described as mustard gas keratopathy (MGK). Clinically, MGK is characterized by ocular inflammation, loss of the epithelial barrier, recurrent epithelial erosions, epithelial-stromal separation, haze/fibrosis, and neovascularization in the cornea [5,7,8,10,12,13,14,15,16,17,18]. To date, no effective and safe treatment for MGK exists despite multiple uses of mustard gas over the last 100 years [19]. This can be attributed to the lack of a clear understanding of the molecular mechanisms involved in MGK.

The cornea provides two-thirds of the light refraction required for vision and protects eye tissues from toxins. Healthy corneas must remain transparent and avascular to deliver sharp vision [20,21]. The cornea has three primary cell layers: the epithelium (outermost), the stroma (middle), and the endothelium (innermost) [22,23,24,25]. The stromal layer constitutes the bulk of corneal thickness (~90%). This layer comprises keratocytes surrounded by lamellae of collagen bundles, extracellular matrix (ECM), and proteoglycans [22,26,27,28,29,30,31]. Keratocytes are the primary cell type in the corneal stroma. They are quiescent and transparent cells with high levels of crystalline structure in their cytoplasm. Keratocytes reside between stromal collagen lamellae and maintain corneal transparency and aid in tissue repair and wound healing [32,33,34,35,36,37,38,39,40,41]. Collagen lamellae make up the structural component of the corneal stroma. After a severe cornea injury, keratocytes transdifferentiate into myofibroblasts [33,42,43]. However, mechanistic insights into SM-induced injury to the cornea remain elusive [19].

Previous studies suggested that SM exposure to the cornea results in myofibroblast formation in the stroma [44,45]. Myofibroblasts are metabolically active, opaque, light-scattering cells that secrete large quantities of ECM, an essential prerequisite for corneal wound healing [28,29,32,33,34]. However, prolonged myofibroblast expression in the stroma leads to scar/haze formation. Studies testing myofibroblast formation after alkali injury showed excessive and persistent myofibroblast formation in early wound healing stages, which is caused by increased levels/activities of TGFβ [25,32,43,46]. Transforming growth factor beta 1 (TGFβ1) plays a central role in corneal dysfunction and haze/fibrosis (opacity) [32]. Our recent pilot rabbit study found increased levels of TGFβ in SM-exposed corneas in vivo [44]. TGFβ1 relays its signal via small mothers against decapentaplegic (SMAD)-dependent or -independent pathways and regulates transcription of ECM remodeling and repair genes [25,47]. In corneal literature, the binding of TGFβ to its receptors leads to the activation of SMADs. There are three major SMAD protein groups: receptor-regulated SMAD2 and SMAD3, common mediator SMAD4, and inhibitory SMAD6 and SMAD7. The TGFβ receptor recruits and phosphorylates SMAD2/3. The phosphorylated pSMAD2/3 complex binds to SMAD4 to translocate into the nucleus. Inhibitory SMAD6 and SMAD7 prevent activation of SMAD signaling by binding to pSMAD2/3 prior to SMAD4 activity [48,49,50,51,52,53].

This study tested a hypothesis that mustard gas exposure activates the TGFβ/SMAD signaling pathway and transdifferentiates stromal fibroblasts to myofibroblasts that produce excessive ECM for an extended period during the corneal wound healing. To test this hypothesis, we determined if mustard gas exposure activated the TGFβ/SMAD signaling pathway using human in vitro and rabbit in vivo models of MGK.

## 2. Materials and Methods

### 2.1. Animals

The Institutional Animal Care and Use Committees of the University of Missouri, Columbia, MO, and MRI Global, Kansas City, MO, USA have approved the study. Rabbits were treated in accordance with ARRIVE and the ARVO Statement for the Use of Animals in Ophthalmology and Vision Research. For this study, we used fifty-four male New Zealand White rabbits purchased from Charles Rivers Laboratories. All rabbits were between 2.5 and 4.0 kg, in good health, and free from any signs of ocular defects at arrival and during the study. Upon delivery, animals were inspected for signs of ill health and quarantined in the animal facility for more than two weeks under the supervision of a veterinarian. All rabbits were fed certified feed and water ad libitum. The rabbits were housed in environmentally controlled rooms at a temperature between 16 °C and 22 °C, with relative humidity of 50% ± 20% with a 12-h light/dark cycle per day.

### 2.2. In Vivo Rabbit SM Studies

Fifty-four eyes of healthy New Zealand White rabbits (2.5–3.0 kg) purchased from the Charles Rivers Laboratories with no clinical ocular symptoms were used. Six eyes for each time point per group were used for statistical analysis based on G-power analysis. All ocular SM vapor and vehicle exposures were performed at MRI Global in Kansas City, MO, and rabbits were transported within 24 h to the University of Missouri, Columbia. Rabbit eyes were divided into three groups: Group 1 (naïve) eyes had no treatment, Group 2 (vehicle) eyes received air vapor, and Group 3 (SM) eyes received SM vapor at a target concentration of 200 mg-min/m^3^ for 8 min [54]. Vapor exposures were given to the whole eye, including the eyelids, conjunctiva, and the globe. SM vapor or vehicle (air vapor) in rabbits was given under anesthesia using ocular vapor goggles following a standard protocol at MRI Global [54]. Animals remained on the shelf unit throughout the exposure period, until a sufficient wash-out of the SM in the goggles was achieved. After that, the goggles were removed, and the eyes were rinsed with a balanced salt solution (BSS). All eyes were subjected to a clinical eye exam and imaging analyses on days 3, 7, and 14 post-SM/air exposure. Thereafter, animals were humanely euthanized and corneal tissues were collected.

### 2.3. Animal Tissue Processing

Following euthanasia, corneas with 2 mm of the sclera were removed by enucleating the eye, puncturing the eye using a number 11 scalpel blade approximately 5 mm posterior to the corneal limbus. A circumferential cut was made to remove the anterior ocular tissues, including the ciliary body and iris. The ciliary body and iris were removed from the cornea using surgical forceps and Westcott scissors under a dissecting microscope (Leica Wild M690, Leica Microsystems Inc., Buffalo Grove, IL, USA). Corneas were cut in two halves: one half was used for histology studies, and the other half was used for molecular studies. The histology half was immediately placed into a mold containing optimal cutting temperature (OCT) compound and snap frozen in a container of 2-methyl butane immersed in liquid nitrogen. Frozen tissues were maintained at −80 °C until sectioning and further evaluation. Tissues were sectioned at 8-μm thickness with a cryostat, mounted on microscopic glass slides (SuperFrost Plus, Fisher Scientific, Waltham, MA, USA), and preserved at −80 °C for subsequent analysis. The molecular half was divided in half to make two quarter corneas. One quarter was used for RNA extraction and cDNA synthesis. One quarter was used for protein analysis.

### 2.4. In Vitro NM Studies

Primary human corneal stromal fibroblasts (hCSF) were generated from human donor corneas purchased from the Saving Sight Foundation, Kansas City, Missouri, using our established protocol [52,55,56,57]. In brief, a #15 surgical blade was used to remove the corneal epithelium and endothelium gently. Afterward, the cornea was cut into eight even pieces and incubated in 60-mm culture dishes with 3 mL Minimum Essential Medium supplemented with 10% fetal bovine serum (FBS) (Thermo Fisher, Grand Island, NY, USA) and grown at 37 °C in humidified 5% CO_2_ incubator. Primary cells were seeded in six-well tissue culture plates at passage three for all in vitro experiments. Each treatment was performed in duplicate, and each experiment was repeated three times (*n* = 6) for high scientific rigor and to evaluate statistical significance. hCSF cells were cultured with or without NM (100 ng/mL) [44]. NM was prepared by reconstituting mechlorethamine hydrochloride (Cat no. 122564, Sigma-Aldrich, St. Louis, MO, USA) in culture media. Additionally, hCSF cells were pretreated with or without SIS3, a SMAD3-specific inhibitor, and exposed to 100 ng/mL of NM. SIS3 (Cat no. 5291, R&D systems, Minneapolis, MN, USA) was solubilized in DMSO. mRNA and protein lysates were collected before treatment, 30 m, 8 h, 24 h, 48 h, and 72 h after treatment.

### 2.5. Real-Time PCR

Corneal tissues were minced in a tissue lyser (TissueLyser LT, Qiagen, Valencia, CA, USA) in RLT buffer (Qiagen, Valencia, CA, USA), and the total RNA was isolated using the RNeasy kit (Qiagen, Valencia, CA, USA) following the manufacturer’s instructions. hCSF cells were collected using RLT butter. The reverse transcriptase enzyme kit was used to synthesize first-strand cDNA (M8291, Promega, Madison, WI, USA). One Step Plus Real-Time PCR system (Cat no. 4309135, Applied Biosystems, Carlsbad, CA, USA) was used for quantitative PCR (qPCR). A 20-μL reaction mixture containing 2 μL cDNA, 2 μL forward and reverse primers (200 nM each), and 10 μL of 2X All-in-One PowerUp SYBR green master mix (Applied Biosystems, Carlsbad, CA, USA) was run at a universal cycle (95 °C for 10 min, 40 cycles at 95 °C for 15 s, and 60 °C for 60 s). Glyceraldehyde 3-phosphate dehydrogenase (GAPDH) was used as an endogenous reference gene. The threshold cycle (Ct) was used to detect the increase in the signal associated with the exponential growth of PCR products during the log-linear phase. ΔCt for each sample was calculated by subtracting the Ct of the target gene from that of the Ct of the endogenous reference gene, and ΔΔCT was calculated by subtracting the ΔCt of the test sample from the ΔCt of the control sample. The relative mRNA expression was calculated using the 2^−ΔΔCt^ method and reported as a relative fold change over the corresponding control values. The amplification efficiency for the qRT-PCR was similar for all templates used, and the difference between linear slopes was less than 0.1. The qPCR was performed in triplicate for each sample, and a minimum of three independent experiments were conducted. Accession numbers and sequences are in Table 1.

### 2.6. Western Blot

hCSF cell lysates were collected in RIPA buffer followed by sonification (3 × 10 s at 50 hz with 30 s gaps). Protein lysate concentrations were quantified following the Bradford quantification method (500006, BioRad, Hercules, CA, USA). 50 µg of protein samples were prepared in 4× Sample buffer (NP0007, Novex ThermoFisher, Waltham, MA, USA) and 10× reducing agent (NP0009, Novex ThermoFisher, Waltham, MA, USA). Then, 30 µL of the prepared sample was loaded onto a precast 12-well NuPAGE 4–12% BT gel. Amersham ECL Rainbow Marker (GERPN800E, Millipore Sigma, Burlington, MA, USA) or SuperSignal M.W. protein ladder (Cat no. 84785, ThermoFisher, Waltham, MA, USA) was loaded. The gel was run and transferred to a nitrocellulose membrane using XCell II Blot Module (E19051, Novex ThermoFisher, Waltham, MA, USA). The nitrocellulose membrane was washed with TBS for 3 × 10 m and blocked with 5% fat-free milk for 1 h at room temperature. Immunostaining was performed using a primary antibody (at recommended dilution) and kept overnight at 4 °C, followed by a 4-h incubation with a secondary antibody (at recommended dilution). The membrane was washed 3× in TBST and incubated in SuperSignal West Femto (34094, Fisher, Pittsburgh, PA, USA) for 10 min. The membrane was then analyzed using a C-Digit digital Western Blot analyzer (C-digit Li-Cor, St, Lincoln, NE, USA). Li-Cor ImageStudioDigits software version 5.2.5 was used for densiometric quantification of western blot bands. This analysis software does not allow tweaking to live capturing and highlights oversaturated images in blue, which prevents quantification of blots. %pSMAD3/SMAD2/3 was calculated by diving pSMAD3 (Cat. No. Sc-517575, Santa Cruz, CA, USA) signal by SMAD2/3 (Cat. No. Sc-133098, Santa Cruz, CA, USA) signal for each timepoint. β-actin (Cat. No. 3700, Cell Signaling, Danvers, MA, USA) signals were compared to the no-treatment group for all timepoints. Then pSMAD3/SMAD2/3 ratios were normalized to respective β-actin expression.

### 2.7. Immunocytochemistry

Cultured cells with and without NM were prepared, post-fixed in 4% PFA at room temperature for 30 m, and blocked with 5% donkey serum for 1 h. Immunostaining was performed using a primary antibody (at recommended dilution) and kept overnight at 4 °C followed by a 4h incubation with a secondary antibody (at recommended dilution). A drop of DAPI antifade Vectashield medium (Cat. No. H1200, Vector Laboratories, Newark, CA) was applied, and tissue sections were mounted with premier coverslips (Thermo Fisher, Waltham, MA, USA). The stained sections were viewed and photographed with a fluorescence microscope (Leica DM 4000B, Leica Microsystems Inc., Buffalo Grove, IL, USA) equipped with a digital camera (SpotCam RT KE, Diagnostic Instruments Inc., Sterling Heights, MI, USA). The number of cell nuclei (DAPI staining) and nuclei with αSMA (Cat. No. M0851, DAKO, Santa Clara, CA, USA) expressions (GFP) was quantified by manually counting six random images taken from culture dishes.

### 2.8. Immunohistochemistry

8-μm thick corneal sections were prepared, postfixed at room temperature for 10 min, and blocked with 5% donkey serum for 1 h at room temperature. Immunostaining was performed using αSMA (Cat. No. M0851, DAKO, Santa Clara, CA, USA) primary antibody (at recommended dilution) and kept overnight at 4 °C, followed by a 4 h incubation with a secondary antibody (at recommended dilution). A drop of DAPI antifade Vectashield medium (Cat. No. H1200, Vector Laboratories, Newark, CA) was applied, and sections were mounted with premier coverslips. The stained sections were viewed and photographed with a fluorescence microscope (Leica DM 4000B, Leica Microsystems Inc., Buffalo Grove, IL, USA) equipped with a digital camera (SpotCam RT KE, Diagnostic Instruments Inc., Sterling Heights, MI, USA).

### 2.9. Statistical Analysis

GraphPad Prism Version 9.2 (GraphPad Software, La Jolla, CA, USA) software was used for statistical analysis. Each experiment was conducted independently with n provided in the test, and the values were expressed as mean ± SD. For statistical analysis, the student’s t-test and two-way analysis of variance (ANOVA) with Bonferroni post-hoc test was used. The value of *p* ≤ 0.05 was considered significant. The sample size was determined using the G*Power (3.1.9.4 software, www.psycho.uni-duesseldorf.de/abteilungen/aap/gpower3, accessed on 19 April 2021) priori power analysis method to achieve α = 0.05; power ≥ 0.9.

## 3. Results

### 3.1. SM Exposure Induces Myofibroblast Formation in Rabbit Cornea In Vivo

#### 3.1.1. Time-Dependent Expression of Profibrotic Genes in Rabbit CORNEAS Exposed to SM In Vivo

**Figure 1:**
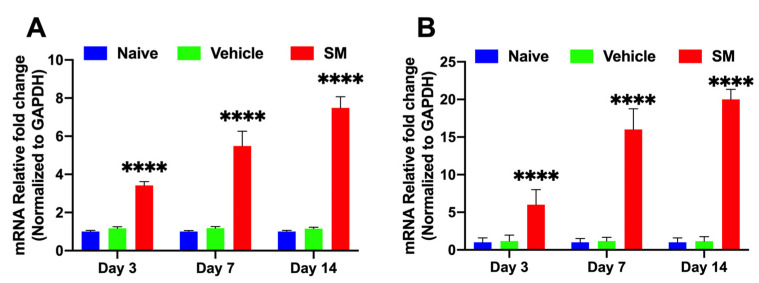
Expression of TGFβ and αSMA mRNA in rabbit corneas in vivo −/+ SM. SM-exposed corneas showed significantly increased mRNA levels of TGFβ (**A**) on day 3 (3.21 ± 0.31; *n* = 6; *p* ≤ 0.0001), day 7 (5.14 ± 0.41; *n* = 6; *p* ≤ 0.0001), and day 14 (7.11 ± 0.19; *n* = 6; *p* ≤ 0.0001) compared to naïve and vehicle corneas. SM-exposed corneas showed significantly increased mRNA levels of αSMA (**B**) on day 3 (7.21 ± 0.51; *n* = 6; *p* ≤ 0.0001), day 7 (16.31 ± 0.71; *n* = 6; *p* ≤ 0.0001), and day 14 (19 ± 0.22; *n* = 6; *p* ≤ 0.0001) compared to naïve and vehicle corneas. (**** = *p* < 0.0001; SM compared to VC).

SM-exposed rabbit eyes showed significantly increased mRNA levels of TGFβ (Figure 1A) on day 3 (3.21 ± 0.31; *p* ≤ 0.0001), day 7 (5.14 ± 0.41; *p* ≤ 0.0001), and day 14 (7.11 ± 0.19; *p* ≤ 0.0001) compared to naïve and vehicle-exposed corneas. Similarly, αSMA, a marker for corneal myofibroblast formation (Figure 1B), was significantly increased on day 3 (7.21 ± 0.51; *p* ≤ 0.0001), day 7 (16.31 ± 0.71; *p* ≤ 0.0001), and day 14 (19 ± 0.22; *p* ≤ 0.0001) compared to the control groups.

#### 3.1.2. Localization of αSMA in Rabbit Corneas Exposed to SM In Vivo

**Figure 2:**
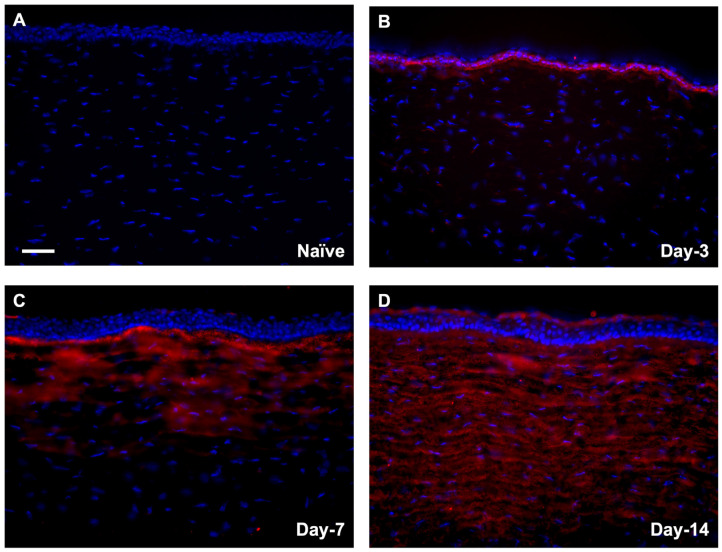
Expression of αSMA protein in rabbit corneas in vivo −/+ SM. SM-exposed corneas had increased staining of αSMA in the corneal stroma in a time-dependent manner compared to naïve (**A**). Day-3 did not have much αSMA staining (**B**) compared to day-7 (**C**) and day-14 (**D**). Day-7 had αSMA staining at the anterior stroma (**C**) and day-14 had αSMA staining throughout the corneal stroma (**D**). (Red = ɑSMA; Scale bar = 100 µM; *n* = 6).

SM-exposed corneas had increased immunostaining of αSMA expression in the corneal stroma in a time-dependent manner. Day-3 corneas had minimal αSMA staining (Figure 2B), which increased significantly on day 7, and day 14. In addition, day-7 had αSMA staining localized in the anterior stroma (Figure 2C), whereas day-14 had αSMA staining throughout the corneal stroma (Figure 2D) compared to the naïve group (Figure 2A).

#### 3.1.3. Time-Dependent Expression of SMAD Signaling Proteins in Rabbit Corneas Exposed to SM In Vivo

**Figure 3:**
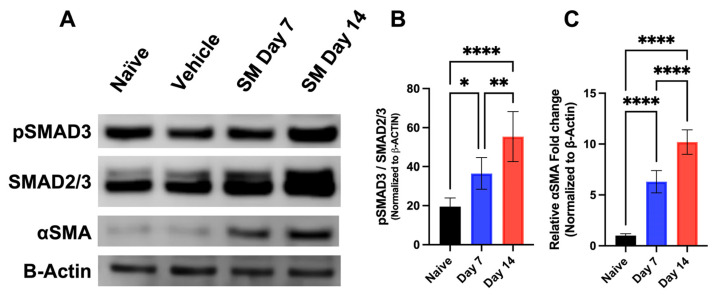
Representative western blots show Time-dependent expression of profibrotic proteins in rabbit corneas exposed to SM in vivo (**A**). SM-exposed corneas had a significant increase in pSMAD3/SMAD2/3 ratio at day 7 (36.51 ± 8.10% vs. 19.49%; *n* = 6; *p* < 0.05) and day 14 (55.36% vs. 19.49%; *n* = 6; *p* < 0.0001) compared to the naïve group (**B**). ɑSMA expression was significantly increased at day 7 (6.31 ± 1.10; *n* = 6; *p* < 0.0001) and day 14 (55.36 ± 12.8; *n* = 6; *p* < 0.0001) compared to NT group. (* = *p* < 0.05, ** = *p* < 0.01, **** = *p* < 0.0001) (**C**).

SM-exposed corneas had a significant increase in phosphorylated SMAD3 to total SMAD (pSMAD3/SMAD2/3) expression at day 7 (*p* < 0.05) and day 14 (*p* < 0.0001) compared to the naïve group (Figure 3). ɑSMA expression was significantly increased at day 7 (*p* < 0.0001) and day 14 (*p* < 0.0001) compared to the naïve group.

### 3.2. NM Exposure Induces Myofibroblast Formation in a Time-Dependent Expression In Vitro

#### 3.2.1. Time-Dependent Analysis of αSMA in hCSFs Treated with NM In Vitro

**Figure 4:**
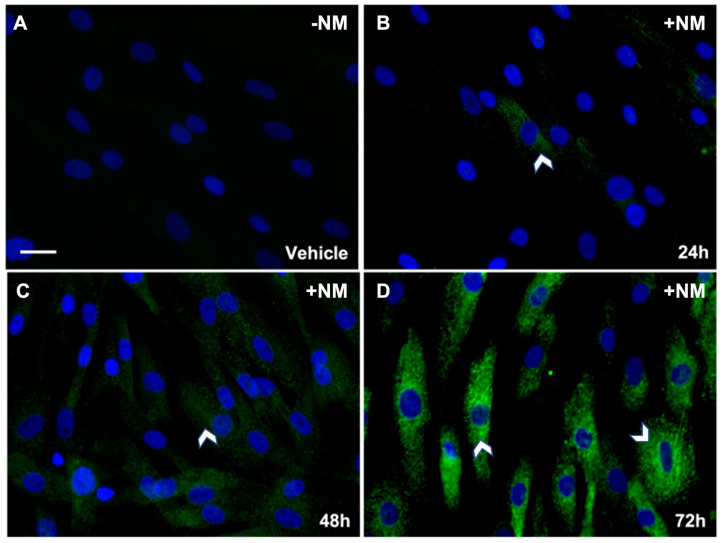
Representative ICC images show time-dependent expression of αSMA in hCSF −/+ NM. NM vapor caused a time-dependent increased expression of αSMA in corneal fibroblasts over 72 h. Low αSMA expression was denoted by weak positive staining at 24 h ((**B**); 10% of cells) and 48 h ((**C**); 55% of cells) post-NM treatment. Intense positive staining represents an increase in αSMA at 72 h ((**D**); 90% of cells) post-NM compared to hCSF with vehicle only ((**A**); 0% of cells). αSMA was stained with GFP (green), and cell nuclei are stained with DAPI (blue). (White arrow heads = ɑSMA; Scale bar = 50 µM; *n* = 3).

For mechanistic studies, we used nitrogen mustard (NM), an analog of SM, in our experiments in vitro. Figure 4 shows a time-dependent increased expression of αSMA in hCSFs grown in −/+ NM for 24 h, 48 h, and 72 h. Low αSMA expression was noted by weak staining at 24 h (Figure 4B) and 48 h (Figure 4C) post-NM treatment compared to the vehicle group. However, intense staining represents an increase in αSMA expression at 72 h (Figure 4D) post-NM compared to the vehicle group (Figure 4A). The marked increase in ɑSMA staining demonstrates corneal fibroblasts are transdifferentiated to myofibroblasts by 72 h after NM treatment.

#### 3.2.2. Profibrotic Protein Analysis of hCSFs Treated −/+ NM In Vitro

**Figure 5:**
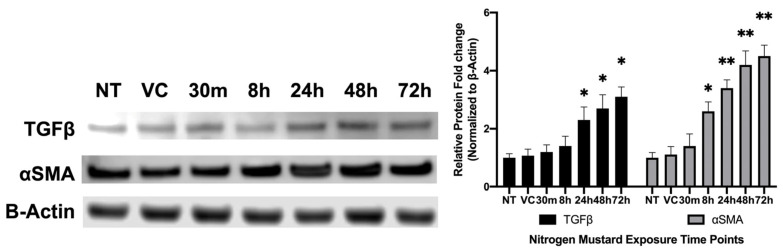
Representative profibrotic protein analysis of hCSF treated −/+ NM in vitro. A significant increase in TGFβ expression in a time-dependent manner at 24 h (2.3 ± 0.45; *n* = 6; *p* < 0.05), 48 h (2.7 ± 0.47; *n* = 6; *p* < 0.05), and 72 h (3.1 ± 0.34; *n* = 6; *p* < 0.05) compared to vehicle control (VC). Quantification of αSMA had a significant increase in a time-dependent manner at 8 h (2.6 ± 0.32; *n* = 6; *p* < 0.05), 24 h (3.4 ± 0.28; *n* = 6; *p* < 0.01), 48 h (4.2 ± 0.48; *n* = 6; *p* < 0.01), and 72 h (4.5 ± 0.38; *n* = 6; *p* < 0.01) compared to VC. (* = *p* < 0.05, ** = *p* < 0.01 compared to VC).

Western blot analysis showed a significant increase in TGFβ expression in a time-dependent manner at 24 h (2.3 ± 0.45; *p* < 0.05), 48 h (2.7 ± 0.47; *p* < 0.05), and 72 h (3.1 ± 0.34; *p* < 0.05) compared to the no-treatment (NT) and vehicle-control (VC) groups (Figure 5). TGFβ did not have a significant increase at 30 m (1.2 ± 0.24) or 8 h (1.40 ± 0.34) compared to the NT and VC groups. Similarly, αSMA expression depicted a significant increase in a time-dependent manner at 8 h (2.6 ± 0.32; *p* < 0.05), 24 h (3.4 ± 0.28; *p* < 0.01), 48 h (4.2 ± 0.48; *p* < 0.01), and 72 h (4.5 ± 0.38; *p* < 0.01) compared to the NT and VC groups (Figure 5). αSMA expression was nonsignificant at 30 m (1.4 ± 0.42) compared to the NT and VC groups. Protein expression and cell morphology were consistent at all time points. Representative images include NT collected at 24 h.

### 3.3. NM-Induced Myofibroblast Formation Involves Activation of SMAD Signaling Pathway

#### 3.3.1. Time-Dependent Expression of Profibrotic Genes in hCSFs Treated with NM In Vitro

**Figure 6:**
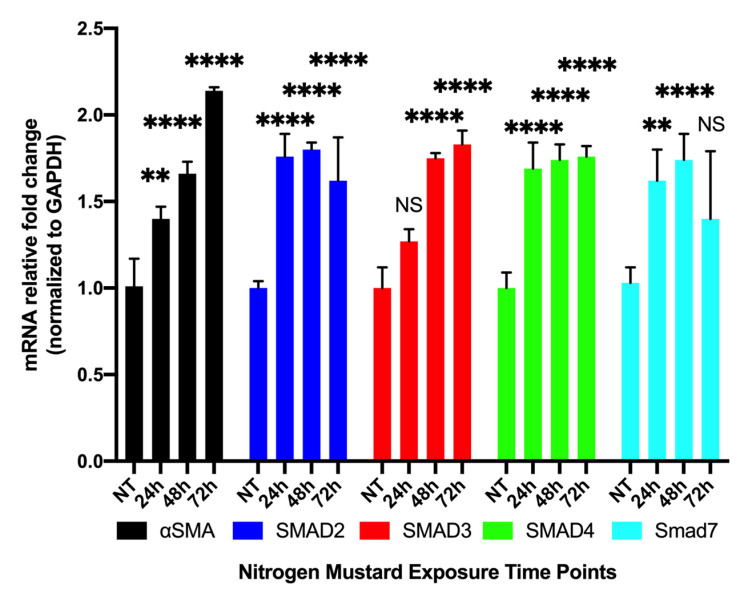
Time-dependent expression of profibrotic genes in hCSF −/+ NM in vitro. qRT-PCR of hCSF in −/+ NM at 24 h, 48 h, and 72 h showed a time-dependent increase in αSMA expression with a significant increase at 24 h (1.4 ± 0.07; *n* = 9; *p* < 0.01), 48 h (1.66 ± 0.07; *n* = 9; *p* < 0.0001), and 72 h (2.14 ± 0.07; *n* = 9; *p* < 0.0001). Likewise, SMAD2 had a significant increase at 24 h (1.76 ± 0.13; *n* = 9; *p* < 0.001), 48 h (1.8 ± 0.04; *n* = 9; *p* < 0.0001), and 72 h (1.62 ± 0.25; *n* = 9; *p* < 0.0001), but this increase plateaued at 72 h. SMAD3 had no significant change at 24 h (1.27 ± 0.07; *n* = 9); however, concentration was significantly increased at 48 h (1.75 ± 0.03; *n* = 9; *p* < 0.0001) and 72 h (1.83 ± 0.08; *n* = 9; *p* < 0.0001). SMAD4 had a significant increase at 24 h (1.15 ± 0.08; *n* = 9; *p* < 0.001), 48 h (1.17 ± 0.13; *n* = 9; *p* < 0.0001), and 72 h (0.93 ± 0.04; *n* = 9; *p* < 0.0001). SMAD7, an anti-fibrotic marker, had a significant increase at 24 h (1.62 ± 0.18; *n* = 9; *p* < 0.01) and 48 h (1.74 ± 0.15; *n* = 9; *p* < 0.0001); however, it did not have a significant increase at 72 h (1.4 ± 0.39; *n* = 9). (NS = not significant, ** = *p* < 0.01, **** = *p* < 0.0001 compared to VC).

qRT-PCR of hCSF in −/+ NM at 24 h, 48 h, and 72 h showed a time-dependent increase in αSMA transcript with a significant increase at 24 h (1.4 ± 0.07; *p* < 0.01), 48 h (1.66 ± 0.07; *p* < 0.0001), and 72 h (2.14 ± 0.07; *p* < 0.0001). Likewise, SMAD2 expression had a significant increase at 24 h (1.76 ± 0.13; *p* < 0.001), 48 h (1.8 ± 0.04; *p* < 0.0001), and 72 h (1.62 ± 0.25; *p* < 0.0001) but plateaued at 72 h. SMAD3 expression had no significant change at 24 h (1.27 ± 0.07); however, concentration was significantly increased at 48 h (1.75 ± 0.03; *p* < 0.0001) and 72 h (1.83 ± 0.08; *p* < 0.0001). SMAD4 expression had a significant increase at 24 h (1.15 ± 0.08; *p* < 0.001), 48 h (1.17 ± 0.13; *p* < 0.0001) and 72 h (0.93 ± 0.04; *p* < 0.0001). SMAD7 expression had a significant increase at 24 h (1.62 ± 0.18; *p* < 0.01) and 48 h (1.74 ± 0.15; *p* < 0.0001); however, it did not have a significant increase at 72 h (1.4 ± 0.39) (Figure 6) compared to the NT group. mRNA expression and cell morphology were consistent at all time points.

#### 3.3.2. Time-Dependent Expression of Profibrotic Proteins in hCSFs Treated with NM In Vitro

**Figure 7:**
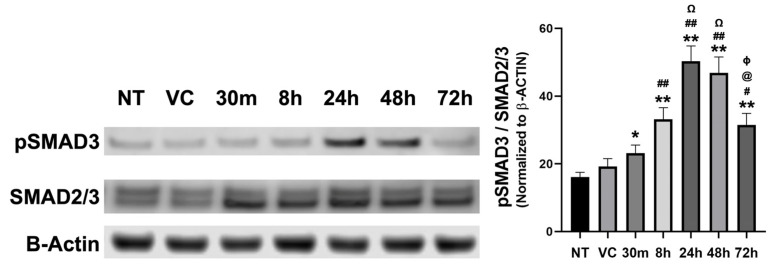
Time-dependent expression of profibrotic proteins in hCSF −/+ NM in vitro. hCSF treated with NM had a significant increase in phosphorylated SMAD3 to total SMAD (pSMAD3/SMAD2/3) ratio at 30 m (23.13%% ± 2.4; *n* = 6; *p* < 0.001), 8 h (33.19% ± 3.4; *n* = 6; *p* < 0.0001), 24 h (50.33% ± 4.5; *n* = 6; *p* < 0.0001), 48 h (46.86% ± 4.7; *n* = 6; *p* < 0.0001), and 72 h (31.49% ± 3.4; *n* = 6; *p* < 0.0001) compared to NT (16.11% ± 1.4; *n* = 6; *p* < 0.001) and VC (19.24% ± 2.3; *n* = 6; *p* < 0.0001) (Figure 4). A significant increase at 8 h (*p* < 0.0001), 24 h (*p* < 0.0001), 48 h (*p* < 0.0001), and 72 h (*p* < 0.001) compared to 30 m. A significant increase at 24 h (*p* < 0.0001) and 48 h (*p* < 0.0001) compared to 8 h. A significant decrease at 72 h (*p* < 0.0001) compared to 24 h and 48 h (* = *p* < 0.001, ** = *p* < 0.0001; NM timepoint compared to NT and VC, # = *p* < 0.001, ## = *p* < 0.0001; NM timepoint compared to 30 m, Ω = *p* < 0.0001; NM timepoint compared to 8 h, @ = *p* < 0.0001; NM timepoint compared to 24 h, ɸ = *p* < 0.0001; NM timepoint compared to 48 h). The data was calculated as the percentage of Smad3 phosphorylation to total SMAD2/3 content.

hCSFs treated with NM had a significant increase in the phosphorylated SMAD3 to total SMAD (pSMAD3/SMAD2/3) ratio at 30 m (23.13%% ± 2.4; *p* < 0.001), 8 h (33.19% ± 3.4; *p* < 0.0001), 24 h (50.33%% ± 4.5; *p* < 0.0001), 48 h (46.86% ± 4.7; *p* < 0.0001), and 72 h (31.49% ± 3.4; *p* < 0.0001) compared to NT (16.11% ± 1.4; *p* < 0.001) and VC (19.24% ± 2.3; *p* < 0.0001) (Figure 4). A significant increase at 8 h (*p* < 0.0001), 24 h (*p* < 0.0001), 48 h (*p* < 0.0001), and 72 h (*p* < 0.001) compared to 30 m. There was a significant increase at 24 h (*p* < 0.0001) and 48 h (*p* < 0.0001) compared to 8 h and a significant decrease at 72 h (*p* < 0.0001) compared to 24 h and 48 h (Figure 7).

In addition to an increase in the pSMAD3/SMAD2/3 ratio, hCSF treated with NM had a significant increase in SMAD3 phosphorylation (pSMAD3) at 8 h (1.57 ± 0.23; *n* = 6; *p* < 0.05), 24 h (2.40 ± 0.18; *n* = 6; *p* < 0.01), 48 h (2.70 ± 0.24; *n* = 6; *p* < 0.01), and 72 h (1.78 ± 0.39; *n* = 6; *p* < 0.05) compared to the VC. However, pSMAD3 was reduced at 72 h compared to 48 h. Additionally, the total amount of SMAD was significantly increased at 8 h (1.52 ± 0.23; *n* = 6; *p* < 0.05), 24 h (1.89 ± 0.42; *n* = 6; *p* < 0.01), 48 h (2.90 ± 0.31; *n* = 6; *p* < 0.01), and 72 h (1.78 ± 0.39; *n* = 6; *p* < 0.05). Similar to pSMAD3, the total SMAD2/3 did not have as great of an increase at 72 h compared to 48 h.

### 3.4. Modulation of NM-Induced SMAD Expression by Specific Inhibitor In Vitro

**Figure 8:**
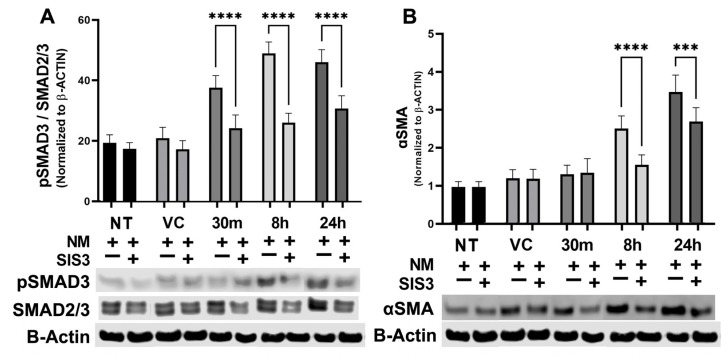
Modulation of NM-induced SMAD expression by SIS3, a SMAD3-specific inhibitor in vitro. hCSFs pretreated with SIS3, a SMAD3-specific chemical inhibitor, to study the functional role of SMAD signaling in myofibroblast formation. SIS3 significantly reduced pSMAD3/total SMAD ratio compared to NM only at 30 m (22.11% ± 3.8 vs. 33.75% ± 3.5, *n* = 6, *p* < 0.0001), 8 h (23.72% ± 2.7 vs. 43.60% ± 3.3, *n* = 6, *p* < 0.0001), and 24 h (27.75% ± 3.7 vs. 41.12 ± 3.6, *n* = 6, *p* < 0.0001) (**A**). The decrease in pSMAD/SMAD2/3 ratio was correlative to ɑSMA expression changes. SIS3 significantly reduced ɑSMA expression compared to NM only at 8 h (1.59 ± 0.27 vs. 2.57 ± 0.34, *n* = 6, *p* < 0.0001) and 24 h (2.76 ± 0.37 vs. 3.56 ± 0.45, *n* = 6, *p* < 0.001) (**B**). (*** = *p* < 0.001, **** = *p* < 0.0001) The data was calculated as the percentage of SMAD3 phosphorylation to total SMAD2/3 content.

Next, we pretreated hCSFs with SIS3, a SMAD3-specific inhibitor, to study the functional role of SMAD signaling in myofibroblast formation. SIS3 significantly reduced the pSMAD3/total SMAD ratio compared to NM only at 30 m (22.11% ± 3.8 vs. 33.75% ± 3.5; *p* < 0.0001), 8 h (23.72% ± 2.7 vs. 43.60% ± 3.3; *p* < 0.0001), and 24 h (27.75% ± 3.7 vs. 41.12 ± 3.6; *p* < 0.0001) (Figure 8A). The decrease in the pSMAD/SMAD2/3 ratio was correlative to ɑSMA expression changes. SIS3 significantly reduced ɑSMA expression compared to NM only at 8 h (1.59 ± 0.27 vs. 2.57 ± 0.34; *p* < 0.0001) and 24 h (2.76 ± 0.37 vs. 3.56 ± 0.45; *p* < 0.001) (Figure 8B).

## 4. Discussion

Sulfur mustard gas (SM) is a potent alkylating agent that causes haze formation upon contact with the eye [58]. Currently, the mechanism of action used by SM to generate corneal haze/fibrosis in the stroma is unknown. Haze results from residential keratocytes transdifferentiating into opaque myofibroblasts (EMT) that produce excess extracellular matrix and persist after physiological wound healing has terminated. Myofibroblasts are absent in a healthy functioning cornea [44,55,59]. Various studies have shown SM has a multifactorial cause of damage, including oxidative stress, inflammation, cell death, and dysregulated signaling pathways [44,45]. In the present study, rabbit corneas exposed to sulfur mustard vapor in vivo showed significantly increased mRNA levels of TGFβ and αSMA on day 3, day 7, and day 14. IHC staining for αSMA in tissue sections confirmed increased αSMA expression and myofibroblast formation in the corneal stroma.

Western blot analysis of SM-exposed rabbit corneas in vivo were used to identify the SMAD signaling pathway as a mechanism for SM-induced myofibroblast formation. Protein analysis showed a significant increase in the pSMAD3/SMAD2/3 ratio and αSMA levels at day 7 and day 14 after SM exposure, thus demonstrating mustard gas exposure caused excessive myofibroblast accumulation in rabbit corneas. Further, a correlative time-dependent increase in SMAD signaling was present after SM exposure in vivo. This expression is analogous to our previous findings suggesting sulfur mustard-induced opacity and edema on day 7, followed by haze/fibrosis on day 14 [58]. Therefore, SMAD signaling was further examined as a possible mechanism for SM-induced corneal myofibroblast in vitro.

Human corneal stromal fibroblast cells (hCSFs) were used to establish TGFβ/SMAD signaling as a molecular pathway for myofibroblast formation. hCSFs were treated with nitrogen mustard (NM), a SM analog used in the lab, in a time-dependent manner in vitro. A significant increase in protein and mRNA levels of αSMA at 24 h, 48 h, and 72 h in were observed in hCSFs, which signifies the formation of myofibroblasts [60]. Additionally, a significant increase in mRNA levels of SMAD2, SMAD3, SMAD4, and SMAD7 was observed in hCSFs. SMAD2, SMAD3, and SMAD4 are described as profibrotic components in corneal wound healing [25]. In addition to keratocyte transdifferentiation to myofibroblasts, the phosphorylation of SMAD3 has also been related to increased ECM production at early time points [25]. Thus, a future study examining the expression of collagen and other ECM would further our understanding of mustard gas-induced fibrosis. Conversely, SMAD7 is an inhibitory member of the SMAD family that prevents the SMAD2/3 complex from binding to SMAD4 and entering the nucleus. Therefore, the increase in SMAD7 at 24 h and 48 h may have a regulatory role in preventing myofibroblast formation. However, a decrease in SMAD7 mRNA was seen at 72 h, which may result from a downstream repressor protein blocking new SMAD7 transcription.

Western blot analysis of hCSFs treated with NM showed a significant time-dependent increase in TGFβ and αSMA protein levels from 24 h to 72 h. SMAD activity was analyzed by testing the pSMAD3 to total SMAD (pSMAD3/SMAD2/3) ratio in a time-dependent manner in vitro. The pSMAD/SMAD2/3 ratio was significantly increased at all timepoints following NM exposure. A time-dependent change can be seen from 30 m to 48 h; however, a slight decrease can be seen at 48 h compared to 72 h. This decrease may result from all hCSFs being converted to myofibroblasts, as seen by ICC staining of ɑSMA by 72 h. In addition, a decrease in pSMAD3 and total SMAD2/3 can be seen at 72 h, which might be due to ubiquitination/degradation.

Further analysis with a SMAD3-specific inhibitor, SIS3, validated the role of SMAD3 and SMAD signaling as a predominant pathway following mustard gas injury. SIS3 caused a significant reduction in pSMAD3/SMAD2/3 from 30 m to 24 h and αSMA protein expression from 8 h to 24 h compared to the NM only group. An interesting finding was the increase in total SMAD and pSMAD3 even after the use of a chemical inhibitor. These results suggest that the synthesis of new SMAD proteins maybe a unique property of mustard gas keratopathy due to the dual nucleophilic chemical property of the mustard gas. For other alkylating agents, such as sodium hydroxide (NaOH), it typically takes 72 h post-exposure for 80–90% of cells to become myofibroblasts [57]. However, NM caused almost all cells to become myofibroblast between 48 h and 72 h. Another possibility for the continued increase in SMAD2/3 and pSMAD3 is the crosstalk between other signaling pathways driven by mustard gas toxicity. Thus, SIS3 showed the profibrotic cascade of the SMAD family being a dominant pathway in myofibroblast formation compared to inhibitory SMAD7 in hCSF treated with NM. Future RNAseq or luciferase assay studies would provide insight into the observed disconnect between mRNA production and protein translation.

## 5. Conclusions

We conclude that TGFβ/SMAD signaling is a predominant pathway for myofibroblast formation in the cornea exposed to mustard gas toxicity.

## Figures and Tables

**Table 1:** List of accession numbers and sequences used for qRT-PCR.

Target Gene	Species	Accession #	Forward Sequence	Reverse Sequence
GAPDH	rabbit, human	NM_002046.3	GCCTCAAGATCATCAGCAATGCCT	TGTGGTCATGAGTCCTTCCACGAT
αSMA	rabbit, human	NM_001613	AAGATCCTGACTGAGCGT	CAAAGTCCAGAGCGACATAG
Smad 2	human	XM_002713521.3	CGAAACGCCACAGTAGAA	GCACTATCACTTAGGCACTC
Smad 3	human	NM_001145102.2	CCCAGAGCAATATTCCAGAG	GTCCATGCTGTGGTTCAT
Smad 4	human	XM_002713541.3	TGGATGTTCAGGTAGGAGAG	CTGTGGACATTGGAGAGTTG
Smad 7	human	XM_017344169.1	ATCACCTTAGCCGACTCT	GCACAGCATCTGGACAAT
TGFβ1	rabbit, human	NM_003242	CGACTACTACGCCAAGGA	GAGAGCAACACGGGTTCA

## Data Availability

Data to share for research use shall be made available upon request.
